# Development of molecularly imprinted vegetarian membranes (MIP-VMs) for oral tissue repair and regeneration

**DOI:** 10.3389/fbioe.2026.1741076

**Published:** 2026-06-16

**Authors:** Molly J. Pritchard, Sharon. R. Oyhanart, Xiaohan Ma, Seyta Diop, Jonathan C. Knowles, Alessandro Poma, David Y. S. Chau

**Affiliations:** Division of Biomaterials and Tissue Engineering, UCL Eastman Dental Institute, London, United Kingdom

**Keywords:** animal-free, biomaterials, biotechnology, membrane, molecular imprinting, regenerative medicine, scaffold

## Abstract

**Introduction:**

Periodontitis-related tooth loss remains inadequately managed, largely due to high dental implant failure rates associated with poor osseointegration. While commercial membranes (i.e., Bio-Gide®, considered the gold standard in dentistry) are widely used to facilitate guided bone regeneration (GBR), their animal-based origin presents ethical, procurement, and accessibility concerns. This study combines a traditional tissue engineering approach to material development with molecular imprinting chemistry to develop a novel, animal-free, recognition-capable biomaterial.

**Methods:**

Icariin, a bioactive flavonoid compound implicated in enhanced osseointegration and tissue regeneration, was imprinted onto eggshell membrane (ESM), onion epidermis (OE) and tomato exocarp (TE) via crosslinking, to evaluate imprinting efficiency and functional performance. The resulting molecularly imprinted vegetarian membranes (MIP-VMs) were characterised by scanning electron microscopy (SEM), water contact angle (WCA), dynamic mechanical analysis (DMA), and icariin binding assays, followed by biocompatibility assessment using human gingival fibroblasts.

**Results:**

Among the tested formulations, the tannic acid (TA)-crosslinked molecularly imprinted ESM (MIP-ESM) demonstrated the most favourable overall performance, exhibiting enhanced stiffness (Young’s modulus increased from 8.5 MPa to 14.0 MPa), increased hydrophilicity (p < 0.01), and improved cytocompatibility (p < 0.05). Notably, the MIP-VMs exhibited several properties comparable to Bio-Gide®, supporting their potential as accessible alternative membrane materials for GBR and other clinical applications.

**Discussion:**

Overall, these results demonstrate the feasibility of generating functional tissue-engineering scaffolds from animal-free materials and present a simple strategy for imprinting membranes with therapeutic agents, highlighting their translational potential in regenerative medicine.

## Introduction

Periodontitis is a chronic inflammatory disease of the gingiva caused by the accumulation of bacteria and debris between the gumline and teeth (Department of Health & Social Care, NHS, England, n.d.). Severe periodontitis results in progressive destruction of the alveolar bone, which may result in tooth loss and is reported to affect approximately 10%–15% of the global adult population, disproportionately affecting disadvantaged socioeconomic patients (Sgolastra et al., 2015). In essence, tooth loss has profound physiological effects including chewing and speech difficulties, and has an acute impact on the patient’s mental health with the corresponding initial trauma triggering progressive alveolar bone resorption, reducing bone height and width and thus destabilising surrounding teeth, ultimately increasing the risk of further tooth loss (International, 2014; Emami et al., 2013).

To address this issue, dental implants offer a long-term solution by providing a stable foundation that supports both functional performance and the aesthetic restoration of the smile (Buser et al., 2017). The long-term success of a dental implant relies on the formation of a direct bond between the alveolar bone-and implant interface through a process called osseointegration (Albrektsson and Johansson, 2001). Since patients with severe periodontitis, however, suffer from significant alveolar bone loss, osseointegration is reduced and therefore the risk of implant failure is significantly raised (Sgolastra et al., 2015; Usui et al., 2021). To overcome this, barrier membranes have been developed to achieve the desired periodontal guided bone regeneration (GBR) to increase the rate of osseointegration by facilitating selective migration and proliferation of osteogenic cells and preventing soft tissue infiltration. Moreover, such a membrane can also prevent ingression of foreign entities such as bacteria (Von Arx and Buser, 2006; Retzepi and Donos, 2010).

Resorbable membranes such as Geistlich Bio-Gide® offer advantages over traditional non-resorbable membranes (e.g., based on polytetrafluoroethylene). They eliminate the need for surgical removal, thereby reducing consequent infection risk and/or localised tissue damage and, as such, are often seen as the “gold standard” within the GBR protocol. However, as these membranes are made with collagen derived from porcine sources, they include limitations ranging from religious incompatibility, disease transmission and high-cost procurement issues (Drzezo, 2020), as well as the additional “consumer choice objection”, i.e., the growing number of patients who adopt vegetarian or vegan lifestyles (Stewart et al., 2021). Moreover, studies have also shown that Bio-Gide® can elicit biomaterial-associated responses including early membrane fragmentation, a mild but persistent inflammatory reaction, and the potential induction of a foreign body response characterised by multinucleated macrophage infiltration during degradation and integration processes (Zhao et al., 2000; Rothamel et al., 2005; Elgali et al., 2017; Ren et al., 2022; Abtahi et al., 2023). These considerations highlight ongoing efforts to develop more ethical, economically accessible, and functionally optimised biomaterials capable of promoting osseointegration and/or facilitating stratification of membrane regeneration and repair.

Herein, extracted membranes derived from the chicken eggshell (ESM, derived from *Gallus domesticus*), onion epidermis (OE, derived from *Allium cepa*) and tomato epidermis (TE, derived from *Solanum lycopersicum*) were explored as alternative candidate biomaterials. It is hypothesised that the natural materials exhibit innate bioactivity which may help further facilitate improved clinical outcomes as well as enabling the bypassing of the ethical, religious and sustainability limitations of animal-derived materials (Jana et al., 2022; Hinrichs-Krapels et al., 2022). Moreover, the ESM contains specific moieties such as fibronectin and heparan sulphate which have been documented to improve periodontal wound healing and therefore could be a leading candidate as an alternative barrier membrane (Pitaru et al., 1991). Although surface adsorption and/or sputtering techniques are often used to improve the functionality of biomaterials to improve their clinical translation, issues such inconsistent molecule loading and innate presence of exogenous factors limit the therapeutic efficacy (Li et al., 2019; Preda et al., 2020; Zaidi, 2020; Hasan et al., 2023). As such, molecular imprinting was employed in this study to introduce recognition sites within the candidate membranes, enabling preferential interaction with the predetermined therapeutic molecule (Erdem et al., 2023). With endpoint clinical applications in mind, i.e., to potentially increase initial and long-term dental implant success, icariin was selected as the target molecule to be imprinted onto the membranes *via* crosslinking. Icariin, a flavonoid, is the active compound of the traditional Chinese medicinal herb *Epimedium* and has emerged as a promising naturally derived bioactive compound that promotes osseointegration (Wang et al., 2016; Zhang et al., 2014; Wang et al., 2012; Mi et al., 2018). Taken together, this study aims to generate proof-of-concept data for the development of molecularly imprinted vegetarian membranes (MIP-VMs) as an emerging set of novel and sustainable biomaterials exhibiting additional recognition functionality that could be exploited for regenerative medical applications.

## Materials and methods

Free-range brown chicken eggs, tomatoes and brown-skinned onions were purchased from a local supermarket (Marks & Spencer, London, United Kingdom). Vancomycin hydrochloride, absolute ethanol (EtOH), acetic acid (99.7%), tannic acid (TA), caffeic acid (CA), glutaraldehyde (GA), foetal bovine serum (FBS), penicillin-streptomycin, L-glutamine, trypsin-EDTA, Triton-X, and phosphate buffered saline (PBS), were all purchased from Merck & Co. (Gillingham, United Kingdom). Bio-Gide® was purchased from Geistlich Pharma United Kingdom. Human gingival fibroblasts [HGnF, (Cat.No. 2620)] were purchased from the ScienCell (ScienCell Research Laboratories, Carlsbad, CA, USA). Icariin (98%) and Dulbecco’s Modified Eagle Medium High Glucose GlutaMAX™ Supplement (DMEM) were purchased from Gibco (Thermo Fisher Scientific, Paisley, United Kingdom). The CellTiter 96® AQueous One Solution Cell Proliferation assay (MTS reagent) and CytoTox 96® Non-radioactive Cytotoxicity Assay (LDH substrate) were purchased from Promega (Southampton, United Kingdom).

### Membrane extraction

**FIGURE 1:**
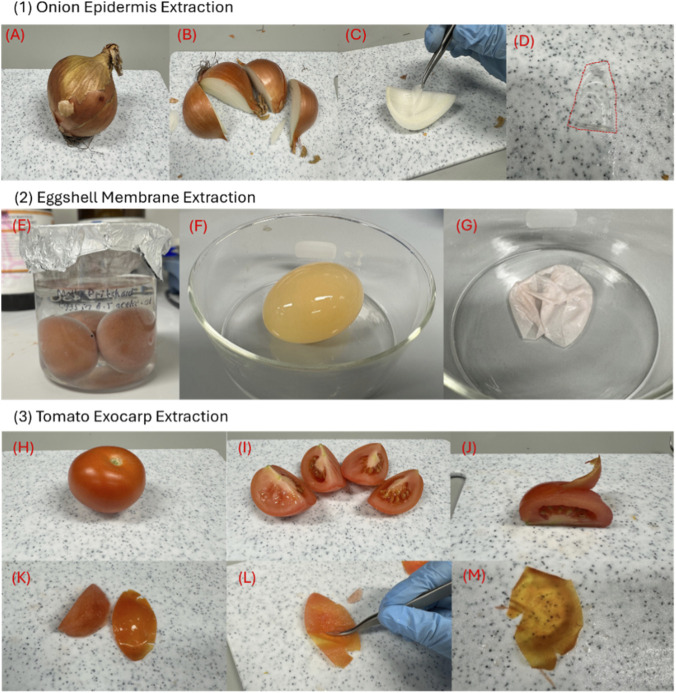
Extraction protocol of the tested biomaterials. The **(1)** OE, **(2)** ESM, and **(3)** TE were isolated as depicted using physical **(1** and **2)** and chemical processes **(3)**. For details regarding the protocol followed for each type of membrane and the labels present in this figure, refer to the body of the text under *Methods-Membrane extraction*.

Membranes were extracted using a range of chemical and physical isolation procedures (Figure 1): brown-skinned onions (A) were quartered (B) and individual layers of the onion were carefully separated using tweezers. The OE was then removed from the inner surface of each layer (C and D). ESMs were isolated from brown chicken eggs by submerging the whole eggs in 0.5 M acetic acid for 120 h at room temperature (RT) (∼19° °C) (E). Eggs were gently stirred daily to ensure the complete exposure/dissolution of the hard CaCO_3_ eggshell by the acid. Thereafter, eggs were removed from the acid, washed extensively with deionised (DI) water before removing the content using a sharp incision (F and G). The TE was isolated from large red tomatoes (H): fruits were thoroughly washed with DI water before being quartered (I) and then the tomato peel was separated from the main body (J and K). Residual tomato flesh was removed by extensive manual scraping using the blunt end of a pair of tweezers (L). Following extraction, all membranes (D, G, M) were thoroughly washed with DI water, stored in the same vehicle at 4 °C and used within 7 days of isolation.

### Molecular imprinting of membranes

Membranes were cut into samples (5 mm × 20 mm and 15 mm × 30 mm). Ten samples of each membrane were submerged for 24 h at RT either in a 50% (v/v) EtOH/H_2_O solution of icariin (0.125 mg/mL), vancomycin (0.27 mg/mL), or neither [non-imprinted membrane]. The template concentrations were selected to promote sufficient template-matrix interactions and enhance recognition site formation whilst avoiding template precipitation (BelBruno, 2018; Hasanah et al., 2021; Szabó et al., 2022; Spivak, 2005). After 24 h, samples were removed and washed thre × e times with 50% (v/v) EtOH/H_2_O at 4 °C. Following template incubation and washing, crosslinker screening was first performed using ESM membranes only. ESM samples were crosslinked for 24 h at RT by immersion in 28 mL of glutaraldehyde [GA, 5% (v/v) in 50% (v/v) EtOH/PBS (1X, pH 7.4)], tannic acid [TA, 0.17 g/mL in 50% (v/v) EtOH/PBS (1X, pH 7.4)], caffeic acid [CA, 0.17 g/mL in 50% (v/v) acetone/PBS (1X, pH 7.4)], or 50% (v/v) EtOH/PBS (1X, pH 7.4) (non-crosslinked control, NCL). Based on the crosslinker screening results obtained with ESM, TA was selected for subsequent membrane preparation and was therefore used for the preparation of OE and TE membranes, as well as for subsequent ESM batches used for physicochemical characterisation and biological testing. Subsequent physicochemical and biological characterisation experiments compared native membranes with their corresponding TA-crosslinked MIP counterparts prepared under these optimised conditions. Following crosslinking reactions, all membranes were removed from the crosslinker solutions and washed three times with 50% (v/v) EtOH/H_2_O at 37 °C. Samples were immediately tested for rebinding assessment or stored in 50% (v/v) EtOH/H_2_O for further characterisation.

### Icariin rebinding assessments

Samples were immersed in 1.5 mL of icariin solution (0.06 mg/mL in 50% (v/v) EtOH/H_2_O). The rebinding concentration was selected to operate within the measurable dynamic range of the assay and to avoid binding site saturation, enabling discrimination between MIP and non-imprinted membranes (Spivak, 2005; Vasapollo et al., 2011; Karrat and Amine, 2024). UV-Vis spectrophotometry was used to measure the absorbance of the solution at 320 nm, at 24 h, using a Unicam UV 500 spectrophotometer (Spectronic, London, United Kingdom). Additional timepoints (5, 48, and 125 h) were also used for further analysis of selected (15 mm × 30 mm) samples, n = 5. The resulting icariin concentration in solution was determined using a calibration curve, and the binding capacity (BC) and imprinting factor (IF) were calculated using Equation 1 and Equation 2, respectively. Rebinding assessments were performed in quintuplicate.

$$\mathrm{Binding Capacity}\left(\%\right)=\frac{C_{i}-C_{f}}{C_{i}}\times 100$$ (1)

where Ci is the initial concentration of icariin in solution (mg/mL) and Cf is the final concentration of icariin in solution (mg/mL) (Harsini et al., 2018)

$$\text{Imprinting Factor}=\frac{Q_{MIP}}{Q_{NIP}}$$ (2)

where Q_MIP_ is the binding capacity of the MIP membrane and Q_NIP_ is the binding capacity of the non-imprinted membrane (Harsini et al., 2018).

### Morphological analysis

The surface morphology of native and MIP-VMs was examined using scanning electron microscopy (SEM) to compare samples and assess post-imprinting modifications. Samples were air-dried, mounted onto 12 mm carbon adhesive tabs attached to 12 mm aluminium pin stubs and sputter-coated with gold/palladium using an E5000 SEM Coating Unit (Quorum Technologies, Laughton, United Kingdom). SEM was conducted using a Zeiss EVO HD microscope (Zeiss, Jena, Germany) with an accelerating voltage of 10 kV and magnifications of ×500 and ×1,000. The surface characteristics of both sides of Bio-Gide® were also imaged for comparison following the same protocol.

### Mechanical analysis

The mechanical characteristics of the native and MIP-VMs, and those of the commercial benchmark Bio-Gide®, were assessed using a film tension clamp and a Discovery DMA850 machine (TA Instruments, Newcastle, United Kingdom). Water-saturated membranes (6 mm × 30 mm) were deformed at a strain ramp rate of 1 mm/min until failure. The strain to failure (StF), force to failure (FtF), and ultimate tensile strength (UTS) were recorded using the dynamic mechanical analyser, while the Young’s modulus (YM) was calculated from the gradient of the initial linear portion of the stress-strain curve. Mechanical testing was performed in quintuplicate at RT.

### Hydrophilicity analysis

Hydrophilicity of the samples was measured by the water contact angle (WCA) using the sessile drop method (Ponoma et al., 2022). The WCA produced on each membrane was measured with an Optical Contact Angle Meter (KSV Instruments, Espoo, Finland) (Figure 2.6). Experiments were performed at RT on blotted native, MIP-VMs and Bio-Gide® samples (10 mm × 10 mm) in quintuplicate. Each value of the contact angle was calculated as an average of three different readings taken under the same conditions.

### Cell viability and cytotoxicity analysis

HGFs at passage 6 were thawed and cultured in supplemented DMEM media (10% fetal bovine serum, 1% penicillin-streptomycin, 2 mM L-glutamine) in Corning T-75 flasks (Corning Life Sciences, Durham, United Kingdom) within a humidified incubator (37 °C, 5% CO_2_). Upon reaching 90% confluence, the cells were detached using standardised protocols with 1x Trypsin-EDTA (0.5 g of porcine trypsin and 0.2 g of EDTA). Membranes were prepared using a 6 mm circular biopsy punch and sterilised with UV radiation for 30 min each side. An aliquot of 30 μL of cells suspension (∼8500 cells) was seeded onto filter paper and membrane samples (native and their corresponding icariin-imprinted MIP counterparts) in a 96 well plate, with three biological replicates per group (membranes obtained from different eggs/tomatoes), each measured in duplicate technical replicates (total observations = 6); membranes of biological replicates were imprinted independently. Cells were incubated for 3 h to facilitate cell attachment to the membranes before 120 μL of complete DMEM media were added to each well. Cells cultured on tissue culture plastic (TCP) were used as controls. Following incubation for defined timepoints in standard cell culture conditions (5% CO2, 37 °C), cytotoxicity and metabolic activity were assessed using the lactate dehydrogenase (LDH) assay (CytoTox96 Non-radioactive Cytotoxicity Assay, Promega) and MTS assay (CellTiter 96®AQueous One Solution Cell Proliferation assay), respectively. For assessing cytotoxicity, a group of cells cultured on TCP were lysed using 1% Triton-X to obtain maximum LDH release. The absorbance of the media-only control at 490 nm was subtracted from all other wells prior to analysis.

To visualize cells viability/death and to support the quantitative analysis, samples were then stained with Live/Dead staining (Invitrogen Life Technologies, Thermo Fisher, Leicester, United Kingdom) according to the manufacturer protocol. The stain was prepared by adding 20 μL of EthD-1 (2 mM) stock solution to 10 mL PBS, combined with 5 mL Calcein AM (4 mM) stock solution. Following the MTS assay, the media was discarded, the samples were rinsed with PBS twice and 100 μL of the stain was added to each sample and incubated at root temperature (∼19 °C) for 20 min protected from light. The viability/death of the cells was visualized using fluorescence microscopy (LEICA Instruments, Milton Keynes, United Kingdom) on Image Capture Pro software. 

Cytotoxicity (%), calculated as the normalised LDH release, where the control is the recording from cells lysed with Triton-X.

### Statistical analyses

The statistical analysis was performed using PRISM (GraphPad Software, version 9) to conduct a one-way analysis of variance (ANOVA) (p < 0.05), followed by *post hoc* Tukey test. The data are presented as the mean ± standard deviation, with statistically significant differences indicated with (*) for p < 0.05, and (**) for p < 0.01.

## Results

### Collagen membrane characterisation

**FIGURE 2:**
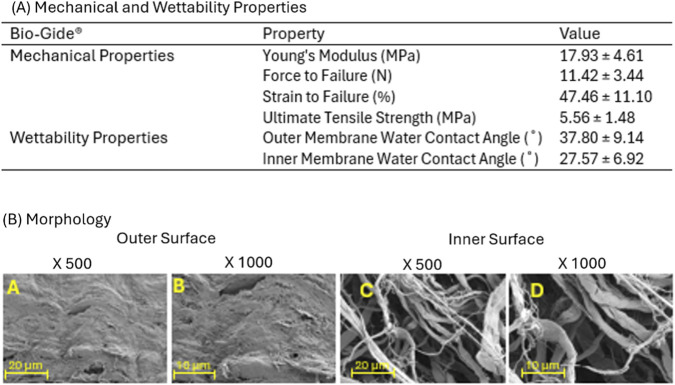
Physical characterisation of Bio-Gide®. **(A)** Quantification of the mechanical and wettability properties of Bio-Gide®, data are presented as the mean ± standard deviation (n = 5). **(B)** Scanning Electron Microscopy (SEM) images of the outer **(A,B)** and inner **(C,D)** surface of Bio-Gide®, magnifications ×500 and X1,000. Scale bars: 20 µm (X500) and 10 µm (X1,000).

Bio-Gide® was initially characterised to identify the required features/parameters of an alternative membrane to suit the translational application performance benchmark as seen in Figure 2.

### Crosslinking reagent optimisation

The efficiency of each crosslinker in enabling successful imprinting was evaluated by assessing the capacity of the material to rebind the target molecule, icariin. Success would be indicated by a significantly higher BC in the icariin-imprinted ESM compared to controls prepared in the absence of template molecules (non-imprinted ESM) or prepared imprinting another template molecule, vancomycin (vancomycin-imprinted ESM).

**FIGURE 3:**
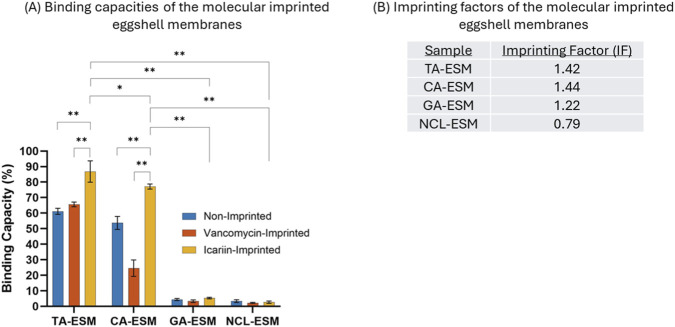
Quantification of icariin imprinting the ESM with different crosslinkers. Tested materials (5 mm × 20 mm) include the TA-crosslinked ESM (TA-ESM), the CA-crosslinked ESM (CA-ESM), GA-crosslinked ESM (GA-ESM), and the native non-crosslinked ESM (NCL-ESM). **(A)** The binding capacity of the non-imprinted (blue), vancomycin-imprinted (red), and icariin-imprinted (yellow) ESMs with different crosslinking reagents at 24 h post rebinding. Data are presented as the mean ± standard deviation and statistical significance was determined by one-way ANOVA with *post hoc* testing (*p < 0.05, **p < 0.01; n = 5). **(B)** The imprinting factors of were calculated as the ratio of the mean icariin rebinding by the icariin-imprinted ESM to that of the corresponding non-imprinted ESM.

The ESM was selected for initial crosslinker screening due to its high collagen content and established susceptibility to chemical crosslinking, providing a robust platform to identify an appropriate crosslinker for the imprinting strategy prior to applying the selected conditions to the plant-derived membranes (Figure 3). Icariin-imprinted ESMs demonstrated higher rebinding of icariin compared to both non-imprinted and vancomycin-imprinted control samples. The non-crosslinked ESM (NCL-ESM) showed no significant difference in icariin binding between icariin-imprinted ESM, vancomycin-imprinted ESM or non-imprinted ESM, and so failed to demonstrate efficacy at imprinting icariin, signifying the need for a crosslinker. The NCL condition served solely as a screening control to confirm that crosslinking was required for imprint stabilisation and was not used in subsequent physicochemical or biological experiments. Significantly more icariin was bound to the icariin-imprinted ESM crosslinked with GA (GA-ESM) compared to the corresponding non-imprinted control (p < 0.01), but the BC in this case was modest, rendering GA inadequate for this specific application. Finally, icariin-imprinted ESMs crosslinked respectively with TA (TA-ESM) and CA (CA-ESM) demonstrated specificity for icariin through significantly higher BCs compared to respective controls (p < 0.01). Both CA and TA therefore successfully enabled the imprinting of icariin onto the ESM. Although the IF of CA-ESM was marginally higher than that of TA-ESM, the TA-ESM exhibited a significantly higher BC than the CA-ESM (p < 0.05). Therefore, TA was deemed the most applicable crosslinking reagent and used for the subsequent material preparation and characterisation experiments. While spectroscopic techniques such as FTIR could provide additional confirmation of crosslinking chemistry, the present study focused primarily on functional screening of crosslinkers based on their influence on imprinting performance. The crosslinking mechanisms of TA, CA, and GA with collagen-based matrices have been extensively characterised in previous studies, including hydrogen bonding and covalent interactions with collagen amine and carboxyl functional groups (Yu et al., 2016; Jafari et al., 2022; Kaczmarek and Mazur, 2020; Wu et al., 2019). In the present study, our objective was not to quantify crosslinking chemistry *per se*, but to screen crosslinkers on the ESM based on their ability to support functional imprinting performance (BC and IF) while maintaining material handling suitability for subsequent characterisation. Accordingly, crosslinker selection was guided by differences in ESM imprinting performance, and TA was advanced for subsequent preparation and characterisation of OE and TE. Detailed spectroscopic characterisation of crosslinking density was therefore considered beyond the scope of the present screening study.

### Surface morphology

**FIGURE 4:**
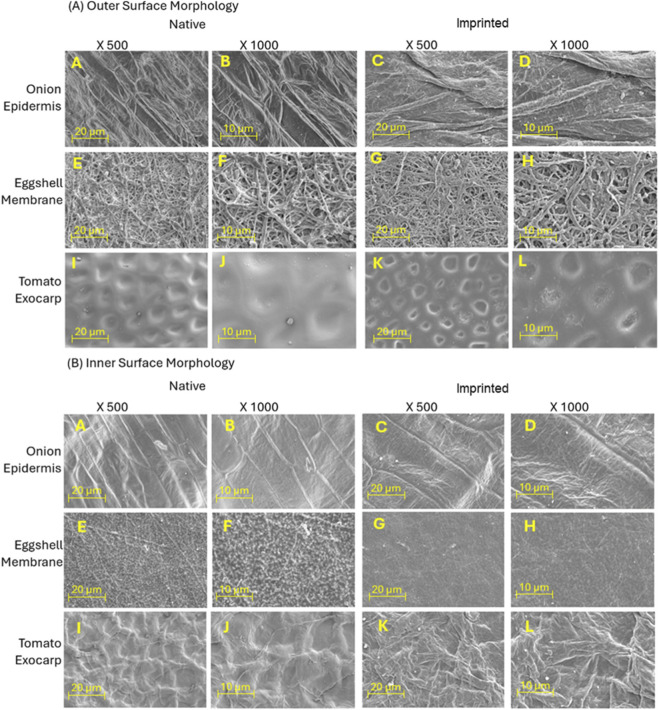
Scanning Electron Microscopy (SEM) images of the native and icariin-imprinted MIP-VMs. **(A)** the outer surface morphology and **(B)** inner surface morphology were imaged at different magnifications (×500 and ×1,000). **(A–D)** OE **(E–H)** ESM, **(I–L)** TE. Images labelled “Native” correspond to untreated membranes, while images labelled “Imprinted” correspond to TA-crosslinked icariin-imprinted MIP-VMs prepared under the optimised conditions identified during crosslinker screening. Scale bars: 20 µm (X500) and 10 µm (X1,000).

Several of the materials investigated exhibited distinct morphological differences between the inner and outer surface (Figure 4). For physicochemical characterisation, comparisons were performed between native membranes and their corresponding TA-crosslinked icariin-imprinted counterparts to assess structural modifications associated with the imprinting treatment. The OE exhibited similar topography, with a smooth and orderly inner surface, and a rougher, more irregular outer surface. The ESM displays a highly interconnected fibrous matrix on the outer surface, and a smooth, dense, and non-fibrous inner layer. Finally, the TE was characterised by a waxy, cuticle-covered outer surface, while the inner side exhibited a non-fibrous collenchymatous layer. Following icariin imprinting, distinct morphological differences were observed across several of the MIP-VMs. The OE outer surface appeared denser and less organised, while the inner surface exhibited a rougher texture and reduced uniformity. The fibrous outer layer of the ESM became less distinct due to the accumulation of additional web-like structures, while the inner surface became denser and smoother. The reduced fibre distinction and increased interfibrillar cohesion observed are consistent with structural compaction associated with TA-mediated crosslinking (Wu et al., 2019; Alves et al., 2022). In contrast, the TE displayed only subtle morphological changes following imprinting: the outer layer developed more defined surface depressions, while the inner layer became rougher with a loss of regular indentation patterns.

### Mechanical properties

A periodontal GBR membrane must withstand physiological mechanical stresses *in vivo*, so the mechanical properties of potential candidate materials were evaluated. TA crosslinking has been shown to enhance the mechanical strength and stiffness of collagenous and other biomaterials by increasing intermolecular cohesion through hydrogen bonding with polar functional groups, resulting in a more compact and mechanically robust network (Jafari et al., 2022; Wu et al., 2019; Bedran-Russo et al., 2009). The stiffness, strength, ductility, and toughness of the native and MIP-VMs were analysed by measuring the Young’s Modulus (YM), Force to Failure (FtF), Strain to Failure (StF), and Ultimate Tensile Strength (UTS), respectively (Table 1).

**TABLE 1:** Mechanical properties of the native and MIP-VMs (n = 5). Data are presented as the mean ± standard deviation and statistical significance was determined by one-way ANOVA with *post hoc* testing (*p < 0.05, **p < 0.01).

​	Young’s modulus (MPa)		Force to failure (N)		Strain to failure (%)		Ultimate tensile strength (MPa)	
**Membrane**	**Native**	**Functionalised**	**Native**	**Functionalised**	**Native**	**Functionalised**	**Native**	**Functionalised**
Onion epidermis	8.09 ± 2.37	25.98 ± 3.50**	0.68 ± 0.15	0.58 ± 0.15	21.38 ± 2.55	21.83 ± 8.83	1.41 ± 0.74	4.64 ± 1.60
Eggshell membrane	8.46 ± 0.47	13.97 ± 2.49**	1.31 ± 0.14	3.65 ± 0.73**	49.83 ± 2.12	82.54 ± 18.50*	5.90 ± 1.38	4.05 ± 0.79*
Tomato exocarp	4.78 ± 1.77	25.04 ± 6.14**	1.89 ± 0.47	1.99 ± 0.59	15.61 ± 1.73	20.08 ± 1.47*	1.54 ± 0.45	4.42 ± 1.75

Overall, the mechanical profile of each material was significantly altered following the TA-mediated crosslinking during the imprinting process, with the MIP-TE displaying a significant increase in StF and YM, suggesting enhanced ductility and stiffness, while the MIP-OE only exhibited enhanced stiffness, indicating restricted effects of the imprinting and crosslinking process. The MIP-ESM, however, showed significantly improved mechanical properties, with greater rigidity (YM), load-bearing capacity (UTS), ductility (StF), and fracture resistance (FtF), highlighting the high susceptibility of ESM to the imprinting process mediated by TA crosslinking. These results are consistent with the role of TA as a natural crosslinker enhancing mechanical integrity (Chen Y. N. et al., 2016; Fan et al., 2018; Lee et al., 2018).

When comparing the physical profiles of the MIP-TE and MIP-ESM, the MIP-ESM was determined as the more flexible material with no statistically significant difference in the load-bearing capacity between the two membranes. However, the MIP-ESM exhibited a significantly higher FtF compared to the MIP-TE, suggesting an enhanced load-bearing capacity despite its lower stiffness, and therefore was determined as the more optimum membrane. Benchmarked against the clinically established Bio-Gide®, the MIP-ESM and MIP-TE demonstrated comparable elastic properties (YM) and tensile strength (UTS), but showed inferior mechanical durability, with a significantly lower FtF (p < 0.01).

### Hydrophilicity analysis

Optimal wettability is critical for facilitating protein and cell adhesion, essential for membrane integration with biological tissues (Arima and Iwata, 2007). Specifically, surface hydrophilicity enhances fibronectin adsorption, a key extracellular matrix protein that supports osteoblast adhesion and proliferation, processes fundamental to successful GBR (Lee et al., 2024). Therefore, the hydrophilicity of the MIP-VMs was assessed.

WCA measurements were used to determine surface hydrophilicity (WCA below 90°) or hydrophobicity (WCA above 90°) (Laurén, 2020). The native and MIP membranes exhibited predominantly hydrophilic tendencies (Table 2).

**TABLE 2:** Quantification of surface hydrophilicity/hydrophobicity properties of native and MIP-VMs, using initial water contact angle measurements (n = 5). Data are presented as the mean ± standard deviation and statistical significance was determined by one-way ANOVA with *post hoc* testing (*p < 0.05, **p < 0.01).

​	Outer membrane water contact angle (°)		Inner membrane water contact angle (°)	
**Membrane**	**Native**	**Functionalised**	**Native**	**Functionalised**
Onion epidermis	83.52 ± 5.40	68.05 ± 3.59**	14.55 ± 2.78	19.49 ± 2.11
Eggshell membrane	68.61 ± 1.77	43.49 ± 4.08**	58.51 ± 2.64	26.30 ± 3.67**
Tomato exocarp	71.40 ± 4.24	71.86 ± 3.23	16.60 ± 3.63	21.92 ± 2.22

The native ESM, TE and OE displayed significantly higher hydrophilicity on their inner surfaces compared to their outer sides (p < 0.01), reflecting the functional polarity of these membranes. Following imprinting, hydrophilicity increased across all samples, evidenced by a reduction in WCA; however, this increase was only statistically significant for the outer surface of the OE and for both surfaces of the ESM (p < 0.01). The high hydrophilicity of both native and MIP-ESM is likely due to the collagen-rich composition, containing numerous polar functional groups (Liu et al., 2019). Additionally, the susceptibility of the ESM to imprinting *via* TA crosslinking further enhanced its wettability, as TA’s abundant hydroxyl groups form hydrogen bonds with water molecules. These data align with previous reports identifying both surfaces of the ESM as hydrophilic (Mensah et al., 2021). Furthermore, SEM evidence of a smoother inner surface may also support in elucidating the observed wettability differences (Wang et al., 2020). Hydrophilicity remained largely unchanged within the MIP-TE, and the membrane retained its natural polarity, exhibiting an inner, highly hydrophilic surface and a more hydrophobic outer surface. This anisotropic wettability reflects the native structure of the TE, comprising a fleshy, water-attracting inner layer and a waxy outer surface composed of hydrophobic cutin molecules (Segado et al., 2016). Excessive hydrophilicity can risk premature membrane collapse due to rapid fluid absorption and can reduce protein adsorption due to competitive water interactions (Zawada et al., 2022). Thus, on comparing the novel candidate materials, the MIP-ESM again provides the most favourable surface properties of a GBR membrane - due to its hydrophilic nature - with respect to both the inner and outer sides.

### Membrane imprinting characterisation and assessment

**FIGURE 5:**
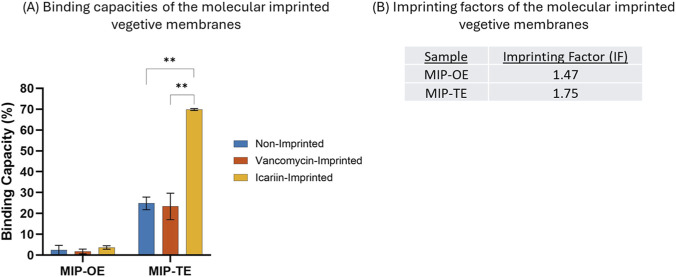
Quantification of molecular imprinting of the vegetable membranes with tannic acid. Tested materials (5 mm × 20 mm) include the MIP-OE and the MIP-TE. **(A)** The binding capacity of the non-imprinted (blue), vancomycin-imprinted (red), and icariin-imprinted (yellow) VMs at 24 h post rebinding. Data are presented as the mean ± standard deviation and statistical significance was determined by one-way ANOVA with *post hoc* testing (*p < 0.05, **p < 0.01; n = 5). **(B)** The imprinting factors of the MIP-VMs were calculated as the ratio of the mean icariin rebinding by the icariin-imprinted membrane to that of the corresponding non-imprinted membrane.

The MIP-TE showed evidence of response to the molecular imprinting process, whereas the MIP-OE showed no evidence of molecular imprinting (Figure 5). The MIP-OE showed low binding capacity (<10%) and no significant improvement in icariin rebinding compared to controls, indicating ineffective imprinting and lack of functional recognition sites. This may be attributed to the cellulose- and pectin-rich composition of the onion cuticle, which provides limited reactive functional groups required for efficient TA crosslinking and stabilisation of template-matrix interactions (Baldwin and Booth, 2022). In contrast, the MIP-TE exhibited a significantly higher BC in the icariin-imprinted membrane compared to both controls at 24 h post rebinding (p < 0.001).

**FIGURE 6:**
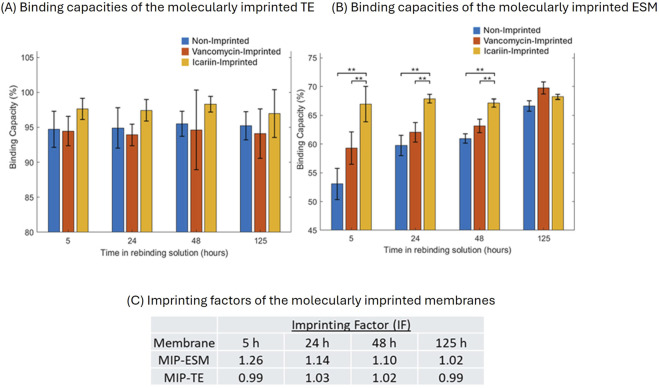
Quantification of molecular imprinting on larger sample sizes. **(A)** The binding capacity of the MIP-TE and **(B)** MIP-ESM, both crosslinked with TA. The binding capacity of the non-imprinted (blue), vancomycin-imprinted (red), and icariin-imprinted (yellow) membranes was measured at 5, 24, 48, and 125 h post rebinding. Data are presented as the mean ± standard deviation and statistical significance was determined by one-way ANOVA with *post hoc* testing (*p < 0.05, **p < 0.01; n = 5). **(C)** The imprinting factors of the MIP membranes were calculated as the ratio of the mean icariin rebinding by the icariin-imprinted membrane to that of the corresponding non-imprinted membrane.

To enhance signal resolution and confirm trends, the membrane dimensions of the MIP-ESM and MIP-TE samples were increased (15 mm × 30 mm) and additional time points were analysed (Figure 6). Under these conditions, a significant increase in icariin binding to the MIP-ESM compared to both controls at 5, 24, and 48 h (p < 0.01), reconfirmed successful icariin imprinting of the ESM. The BC remained consistent over time, suggesting an approach to saturation of the icariin-specific binding sites and stable retention of the molecule. In contrast, the larger TE samples, likely due to their high adsorptive capacity and porous surface structure, exhibited excessive icariin binding across all TE membranes, making the data less interpretable and masking the previously observed improvement of the MIP-TE. However, the TE consistently exhibited a high BC (95%–100%) at all time points, highlighting its ability to bind icariin, albeit in a non-selective manner. Further analysis of IFs using membranes of increased dimensions over time revealed that the MIP-ESM had an IF of 1.26 at 5 h, indicating initial specificity for icariin binding. This gradually declined to 1.02 at 125 h, likely due to increased non-specific surface icariin adsorption by the non-imprinted control, reducing the IF ratio.

Statistically significant increases in icariin binding for the icariin-imprinted MIP-ESM compared to both non-imprinted and vancomycin-imprinted controls at early timepoints support the formation of preferential binding sites during imprinting beyond purely random adsorption. The imprinting factors observed in this study were modest compared to those typically reported for fully synthetic molecularly imprinted polymers, with a maximum IF of 1.26 and values approaching unity at extended incubation times. While higher IF values are frequently reported for rigid, fully synthetic MIP polymer systems, heterogeneous systems frequently exhibit lower apparent selectivity due to contributions from non-specific adsorption and heterogeneous binding site distributions (Spivak, 2005; Umpleby et al., 2004; Chen L. et al., 2016). In biomaterial-based scaffolds and extracellular-matrix-like substrates, structural heterogeneity and porosity can further influence binding behaviour and recognition performance (Neves et al., 2017; Bonatti et al., 2021). In porous collagen-based substrates, non-specific adsorption may contribute substantially to total binding in non-imprinted controls, thereby reducing the calculated IF despite the presence of preferential binding sites. The gradual convergence of IF values towards unity at longer incubation times is therefore consistent with progressive non-specific adsorption and/or saturation effects, phenomena which are recognised to diminish apparent selectivity in heterogeneous systems (Lamaoui et al., 2021).

The high BC of the MIP-ESM is likely associated with effective imprinting facilitated by TA-mediated crosslinking within the collagen-rich ESM matrix, which contains abundant amine, hydroxyl, and carboxyl functional groups capable of interacting with both the template and the crosslinker (Han et al., 2023). Moreover, polyphenols induce steric effects that alter the conformation of the collagen triple helix, leading to a denser and more stabilised collagenous matrix (Madhan et al., 2002). Additionally, ESM collagen is rich in lysine residues that provide free amine groups capable of forming stabilising interactions with TA through hydrogen bonding and Schiff base links, mechanisms that are largely absent in the vegetative membranes (Wu et al., 2024).

The high BC of the MIP-TE is likely primarily due to physical entrapment within its natural porosity, rather than chemical interactions. Although the TE is rich in cutin, abundant with carboxyl and hydroxyl groups, the esterified structural network reduces the accessibility of functional groups required for effective crosslinking and stabilisation of template-matrix interactions (Reynoud et al., 2022). Instead, the high BC of the MIP-TE may be attributed to its nanoporous outer layer and roughened inner surface, features observed *via* SEM, which together increase the surface area available for template interaction compared to the native membrane. Specific rebinding was only apparent in the smaller TE samples. This may reflect greater exposure of surface-located binding sites at higher surface-to-volume ratios, whereas larger samples exhibited dominant bulk adsorption that masked any imprinting effects. Overall, the ESM appeared to be the most responsive to the icariin imprinting strategy, showing clear template recognition and binding efficacy.

Template concentration is a critical parameter in molecular imprinting systems, as template loading directly influences cavity density, affinity distribution, and recognition performance (Spivak, 2005; BelBruno, 2018; Hasanah et al., 2021). The imprinting concentrations used in this study were selected to favour template-matrix interactions and maximise the probability of stable binding site formation while maintaining compatibility with the natural membrane substrates. Although higher template concentrations may theoretically enhance imprint density, icariin exhibits limited aqueous solubility and requires organic co-solvents for dissolution (Szabó et al., 2022). Increasing the ethanol fraction or employing fully organic imprinting conditions was avoided to preserve membrane structural integrity. Furthermore, the rebinding concentration was selected to operate within the measurable dynamic range of the assay and avoid binding site saturation, as sub-saturation conditions are recommended for reliable evaluation of binding behaviour and discrimination between imprinted and non-imprinted membranes (Spivak, 2005; Karrat and Amine, 2024; Vasapollo et al., 2011). These concentrations should therefore be interpreted as methodological optimisation parameters rather than direct reflections of physiological exposure levels.

### Biological activity

**FIGURE 7:**
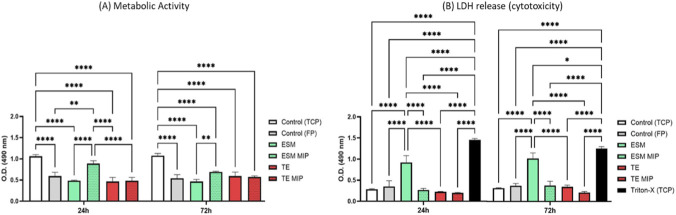
Biochemical assays of human gingival fibroblasts cultured on various membrane scaffolds. **(A)** Metabolic activity normalised to untreated HGFs cultured on filter paper and **(B)** cytotoxicity of HGFs against cells treated with Triton-X was assessed at 1 (blue), 2 (red), and 5 (yellow) days post-seeding. Triton-X represents the positive control for maximum cytotoxicity. Tested materials include the native ESM, MIP-ESM, native TE, and MIP-TE (n = 4). Data are presented as the mean ± standard deviation and statistical significance was determined by one-way ANOVA with *post hoc* testing (*p < 0.05, **p < 0.01).

**FIGURE 8:**
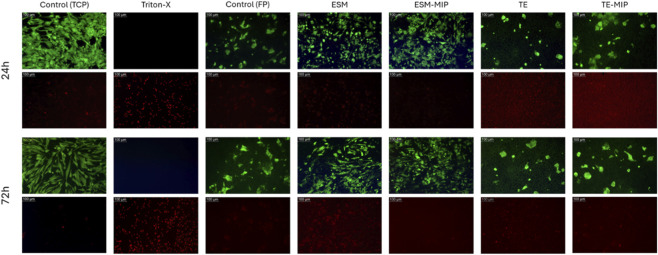
Cell viability and cytotoxicity demonstrated by Live/Dead™ staining. Cells were seeded on control and membrane samples for specific timepoints before being treated with an EthD-1 and calcein AM dual stain, prior to being imaged under a fluorescent microscope: live cells were identified as fluorescent green and dead cells as fluorescent red.

HGFs were cultured to 90% confluency and displayed characteristic fibroblastic morphology, including an elongated, spindle-shape and aligned growth patterns. Cell viability was assessed using Live/Dead staining, whereas metabolic activity and cytotoxicity quanitified using the CellTiter-AQ assay and CytoTox membrane integrity (LDH-release) assays, respectively (Figures 7, 8).

MTS release was normalised against HGFs cultured on filter paper (negative control) to account for baseline metabolic activity and enable comparison of relative cell viability across experimental groups. A significant increase in metabolic activity was observed in the MIP-ESM and MIP-TE against their corresponding native membranes on days 1 and 2 suggesting differences in bioactive icariin availability between membrane types. These findings align with the imprinting data, although a quantitative release profile was not directly measured in this study.

Furthermore, both the MIP-ESM and MIP-TE demonstrated significantly higher cell viability compared to the filter paper control on days 1 and 2 (p < 0.01). The MIP-membranes therefore hold beneficial effects on early cell-material interactions and promote sustained cellular support. The ESM, MIP-ESM, and MIP-TE all exhibited a similar trend of significantly increased HGF activity on day 2 compared to days 1. For the native TE, the peak metabolic activity was seen on day 2 compared to significantly lower activity on day 1 (p < 0.01), which may be attributed to delayed cell attachment and proliferation.

LDH release was measured to validate metabolic activity observed in the CellTiter-AQ assay reflected healthy, proliferating cells rather than metabolically active dying cells. Results were normalised to Triton-X treated cells (positive control), which induced complete cell lysis and maximal LDH release. HGFs cultured on TCP, filter paper, MIP-TE, and native TE showed consistently lower LDH release compared to those cultured on the ESM and MIP-ESM, suggesting some degree of cytotoxicity with the ESM. However, the MIP-ESM significantly reduced LDH release on day 2 compared to the native ESM, indicating imprinting contributes to improved biocompatibility. The MIP-TE did not show a significant reduction in cytotoxicity. This may be attributed to the already low LDH release observed in the native TE membrane, indicating that the cytoprotective effects of icariin were likely unnecessary in this context. Interestingly, the MIP-TE showed significantly lower LDH release than the TCP control on day 2, highlighting superiority against an optimum surface (p < 0.05).

As reported within the literature, icariin promotes tissue regeneration *via* the enhancement of cell viability and general reduction in cytotoxicity (Mi et al., 2018; Liu and Fang, 2022). These have been found to be in agreement with the data obtained within this study and, most likely due to the activation of PI3K/Akt signalling to support cell survival and free radical scavenging to reduce oxidative stress- a key characteristic of icariin-mediated response (Deng et al., 2017; Zhang et al., 2010). A consistent trend across all but the TCP group, was a significantly reduced LDH release on day 2 compared to day 1. This likely reflects an initial stress response upon seeding and the transition from standard culture conditions (day 1), followed by a recovery phase (day 2) as cells adapt and re-establish membrane integrity. By day 5, LDH levels increased again, which could be attributed to media depletion, accumulation of metabolic waste, or over-confluency leading to necrosis. This trend aligns with the CellTiter-AQ assay results, which demonstrated increased metabolic activity on day 2, indicative of healthy, proliferating cells and, consequently, reduced LDH release.

Although differences in cellular response suggest bioactive icariin availability from the imprinted membranes, a quantitative cumulative release profile was not determined in the present study. BC and IF measurements provide insight into preferential rebinding behaviour, but do not directly quantify release kinetics. Future work will therefore include systematic evaluation of drug release behaviour and kinetic modelling to characterise drug elution profiles under physiologically relevant conditions.

In light of the results presented herein, MIP-VMs, particularly MIP-TE and MIP-ESM, appear to be promising candidates for GBR procedures, exhibiting good cytocompatibility and acceptable physicochemical properties. Notably, MIP-ESM displayed a mechanical tensile profile close to that of the current gold standard for resorbable collagen GBR membranes (i.e., Bio-Gide®), as well as a similar degree of wettability, in addition to a high binding capacity enabled by effective crosslinking with tannic acid. The ESM has previously been regarded as a biocompatible and biodegradable material with low immunogenicity (Chen et al., 2022), and previous work within our group has indicated favourable responses in an *in vivo* model of periodontal defect (unpublished data). Nevertheless, further *in vitro* and *in vivo* investigations will be required to confirm long-term tolerability and regenerative performance of the imprinted membranes. Although statistically significant differences in binding capacity between imprinted and control membranes support a functional imprinting effect, the imprinting factors observed in this study were moderate and declined over extended incubation. This behaviour is consistent with the heterogeneous and porous nature of biomaterial substrates, where non-specific adsorption contributes to baseline binding and may reduce apparent selectivity. The present study focused on functional performance rather than detailed mechanistic characterisation of binding and crosslinking. Adsorption isotherm modelling and advanced characterisation of crosslinking (e.g., *via* FTIR or related spectroscopic techniques), as well as evaluation of binding site affinity and distribution, were therefore considered beyond the scope of this work. Future investigations will aim to systematically evaluate crosslinking density, binding thermodynamics, and release kinetics to further optimise imprint specificity and prospective therapeutic performance. It has been previously suggested that crosslinking can reduce the immunogenicity of collagen-based membranes by masking antigenic markers (Caliari and Harley, 2011; Wang et al., 2015), which may further support the translational potential of this approach *in vivo*. Moreover, additional *in vitro* studies using stem cell populations could provide further insight into whether these membranes possess inherent osteoinductive or regenerative properties.

## Conclusion

The clinical demand for improved barrier membranes for periodontal GBR arises from the limitations of existing animal-derived resorbable and non-resorbable membranes. Based on the data generated within this study, the MIP-ESM crosslinked with TA appeared to be the most promising candidate, owing to its specific icariin rebinding and its favourable physical profile, including consistent hydrophilicity, appropriate flexibility and mechanical strength, and a dual-surfaced topography. Moreover, the biological profiling of these novel icariin-imprinted materials demonstrated potential in gingival tissue compatibility and, in essence, the realisation for regeneration and repair of tissue within the oral cavity. The MIP-ESM therefore represents a promising alternative GBR periodontal barrier membrane, offering a promising platform for selective drug loading and potential future development as a controlled delivery system through molecular imprinting, as well as practical advantages stemming from its abundance, low cost, and ethical sourcing.

## Data Availability

The original contributions presented in the study are included in the article/supplementary material, further inquiries can be directed to the corresponding author.
